# Supplementary material for: Differential coding of reward and movement information in the dorsomedial striatal direct and indirect pathways
Source: Nat Commun. 2018 Jan 26;9:404. doi: 10.1038/s41467-017-02817-1 (PMC5786099; doi:10.1038/s41467-017-02817-1)
Supplement: Supplementary file 1 — Supplementary Information [file 41467_2017_2817_MOESM1_ESM.pdf]

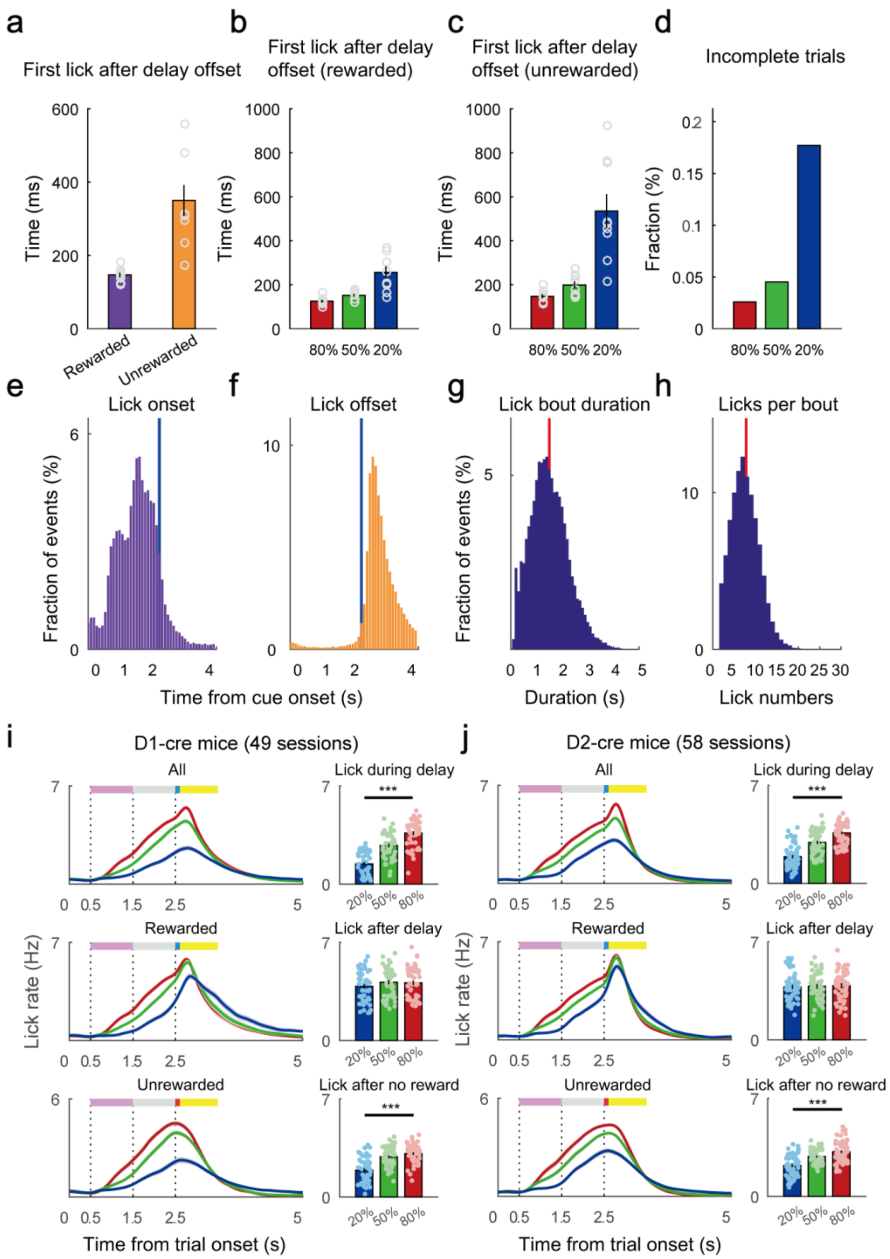

Supplementary Figure 1. Licking behavior. (**a**) The time from delay offset (i.e., from reward delivery in rewarded trials and light onset in unrewarded trials) to first lick. (**b, c**) The time from delay offset to first lick is shown as a function of reward probability for rewarded (**b**) and unrewarded (**c**) trials. The open circles denote individual animal data (five D1-cre and four D2-cre mice). (**d**) The proportion of incomplete trials (no lick between delay offset and next trial

onset) is shown as a function of reward probability (n=107 sessions). (**e-h**) Distributions of lick onset time (**e**), lick offset time (**f**), lick bout duration (**g**), and licks per lick bout (**h**). Most lick onsets occurred before delay offset and most lick offsets occurred after delay offset (i.e., 2 s after cue onset). (**i, j**) Licking behavior of five D1-Cre (**i**) and four D2-Cre (**j**) mice. The filled circles denote individual session data (49 and 58 sessions in i and j, respectively). The same format as in Fig. 1C, D. ***$p < 0.001$ (ANOVA followed by *post-hoc* Tukey's test).

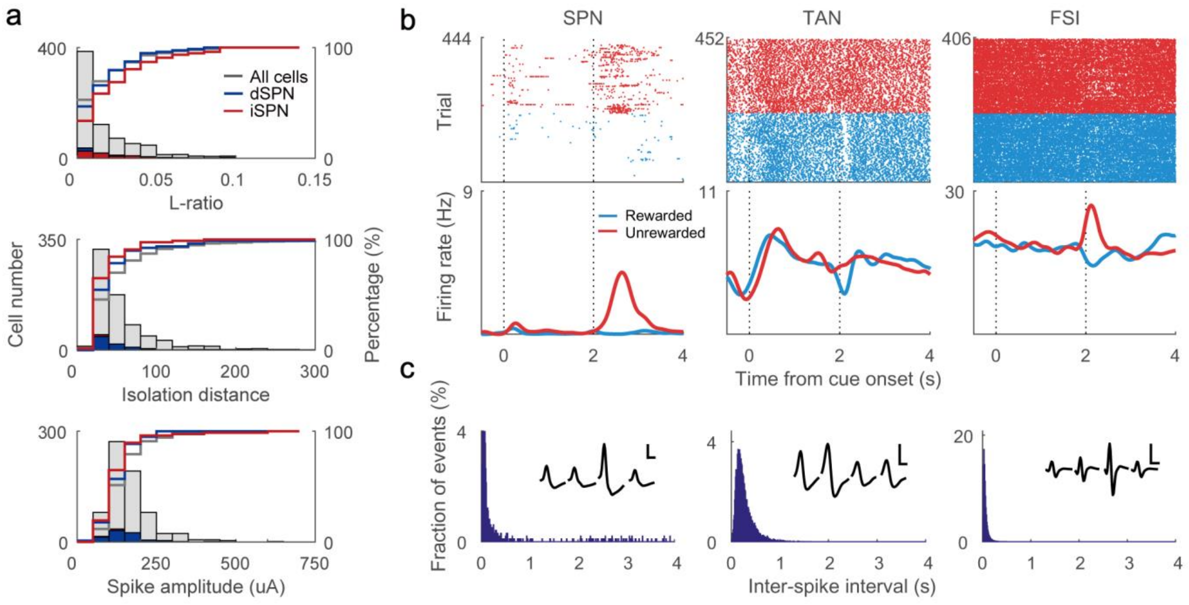

Supplementary Figure 2. Unit isolation parameters and example responses of different types of striatal neurons. (a) Stacked and cumulative histograms for L-ratio (top), isolation distance (middle) and spike amplitude (bottom) of the unit clusters shown in Fig 2g including optogenetically identified D1 (blue) and D2 (red) unit clusters. **(b)** Three representative examples of SPN, TAN, and FSI activity during the probabilistic Pavlovian conditioning task. Top, spike raster plots; bottom, spike density functions (σ=100 ms). Data are grouped into rewarded (blue) and unrewarded (red) trials. Vertical dashed lines indicate cue onset (left) and delay offset (right). **(c)** An inter-spike interval histogram and averaged spike waveforms are shown for each example neuron. Calibration, 250 μs and 50 μV.

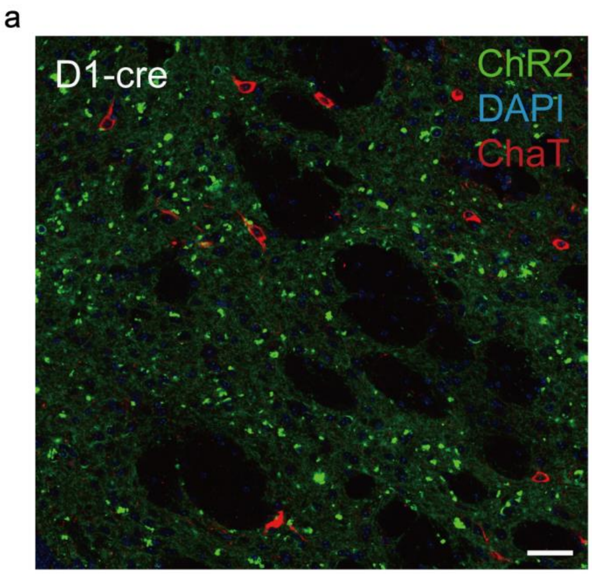

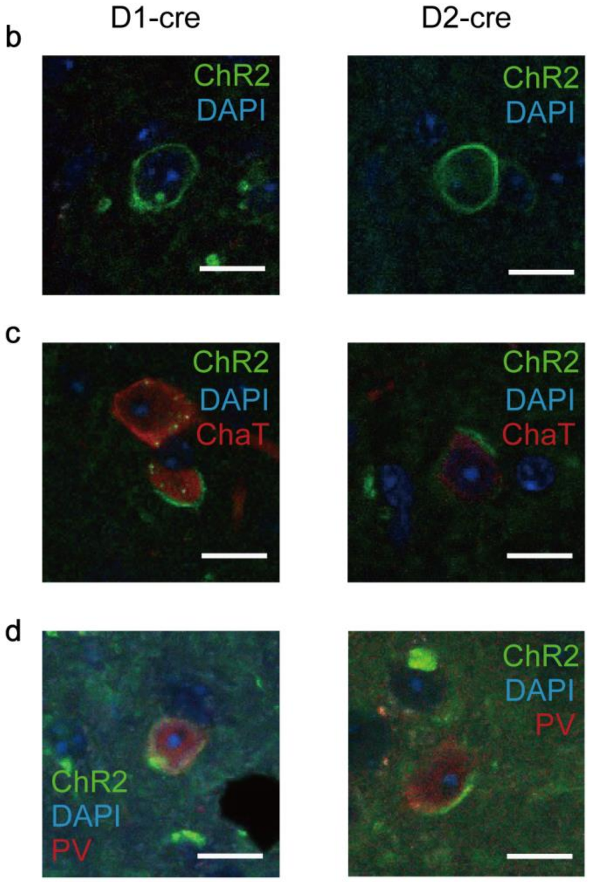

e

Mouse line	Marker	Marker positive cells /number of mice	Co-labeled with ChR2
D1-ChR2	ChaT	197/4	13 (6.6%)
	PV	167/2	2 (1.2%)
D2-ChR2	ChaT	146/4	9 (6.2%)
	PV	80/2	2 (2.5%)

Supplementary Figure 3. Striatal interneurons express D1 and D2 receptors. We performed immunohistochemistry using brain slices obtained from the mice used for unit recording (Fig. 2) or optical stimulation (Fig. 8). **(a)** An example brain section obtained from a D1-Cre mouse showing choline acetyltransferase (ChaT; red) and ChR2 (green) immunolabeling. **(b-d)** Example brain sections obtained from D1-Cre (left) and D2-Cre (right) mice. **(b)** Example striatal neurons expressing ChR2 in the cell membrane. **(c)** Examples of ChaT and ChR2 co-labeled neurons. **(d)** Examples of PV and ChR2 co-labeled neurons. **(e)** Percentages of co-labeled neurons. Scale bar, 50 μM (**a**) and 10 μm (**d-e**).

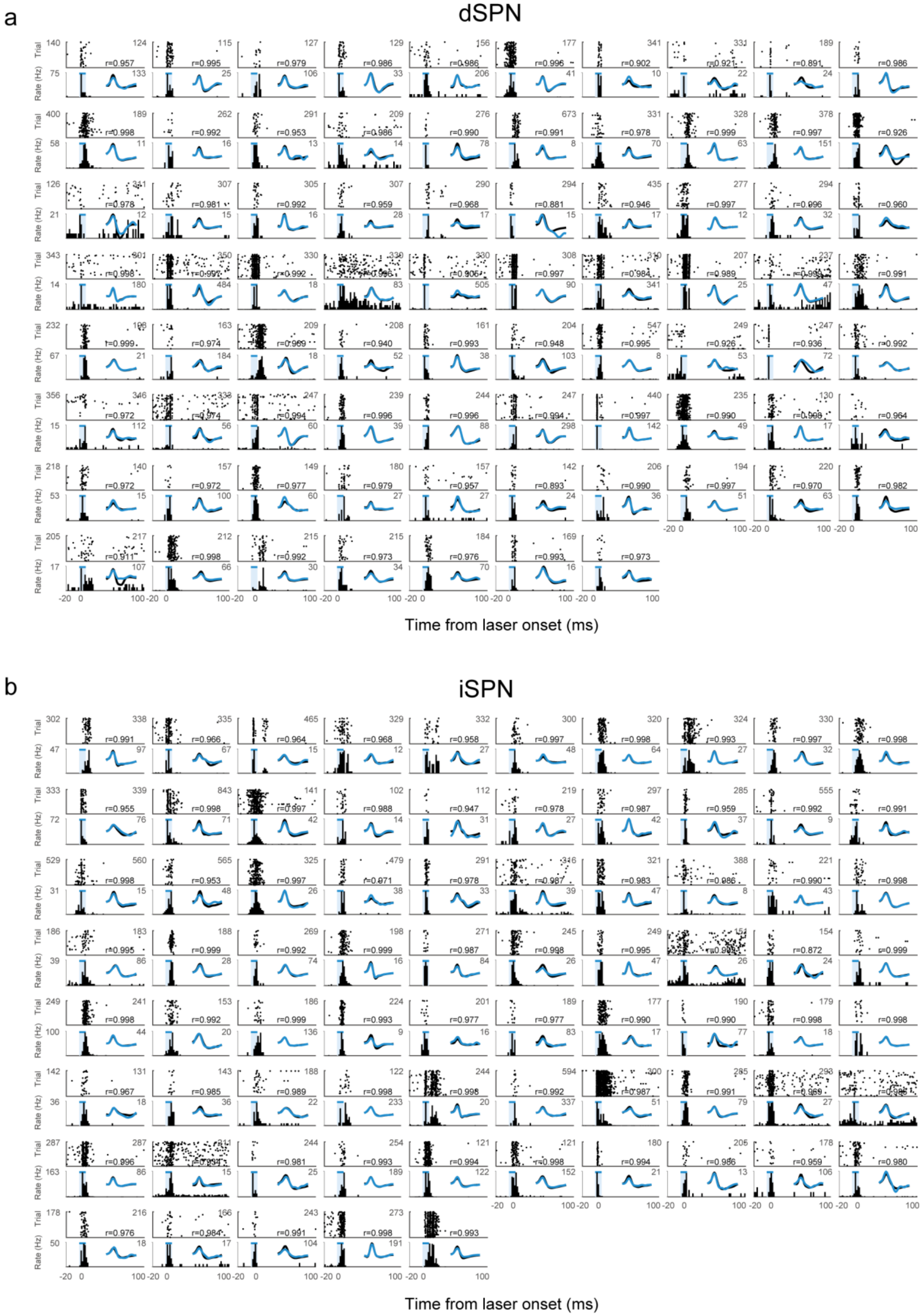
a
dSPN
Trial
Rate (Hz)
Time from laser onset (ms)
b
iSPN
Trial
Rate (Hz)
Time from laser onset (ms)

Supplementary Figure 4. Responses of all optogenetically identified dSPNs and iSPNs to laser stimulation. A raster plot, a PSTH, and averaged waveforms of spontaneous (black) and laser-driven (blue) spikes are shown for each neuron. *r*, correlation between laser-driven and spontaneous spike waveforms (spike waveform similarity). Blue rectangles indicate laser stimulation (10 ms).

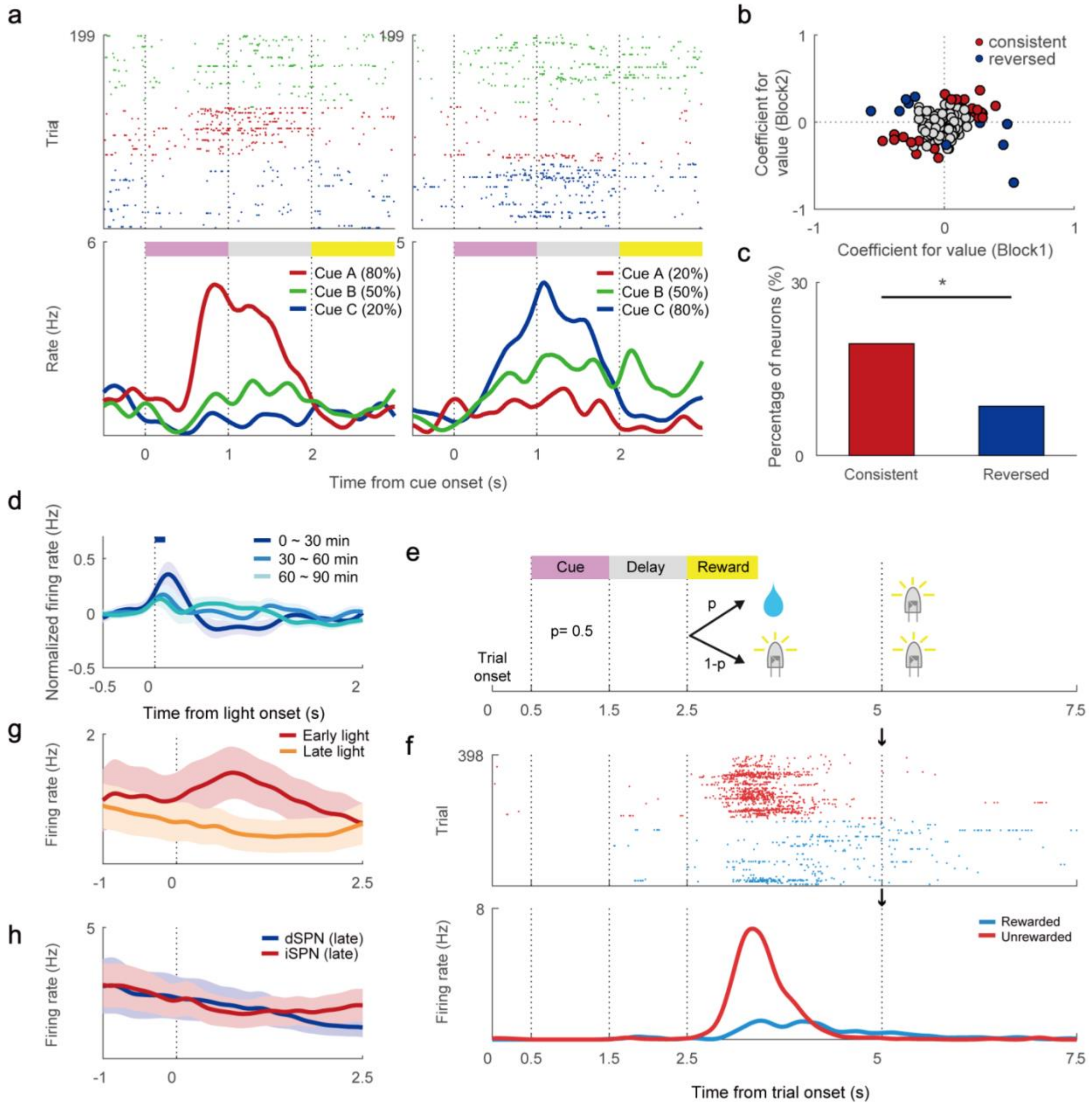

Supplementary Figure 5. Control experiments for odor- and light-related neural activity. We performed additional physiological experiments in a separate group of mice (two D1-Cre and one D2-Cre mice; ChR2 expressions and optical probe/recording locations confirmed by histological examination as in Figure 2) to test sensory cue-related SPN neural activity. **(a-c)** To test the possibility that seeming value-dependent striatal neural activity might actually represent odor cue-dependent neural activity, we recorded dorsomedial striatal neural activity in a daily reversal task. The cue-reward probability relationship was reversed (i.e., reward probabilities were changed from 20, 50, and 80% to 80, 50, and 20% for a given set of cues) across two

blocks (150-200 trials per block) in each day, and those sessions with significantly different mean lick rates across the three cue conditions (one-way ANOVA, $p<0.05$) during the cue/delay period for both blocks were included in the analysis (19 sessions, 129 units). **(a)** An example SPN showing similar activity relationships with value before and after reversal (spike density functions, σ=100 ms). **(b)** Group data. The scatter plot shows regression coefficients for value (*eq*. 1) before (abscissa) and after (ordinate) reversal. Colored circles indicate those units significantly responsive to value (*eq*. 1) during the first block. Red and blue indicate the same and opposite signs of the regression coefficients across the two blocks, respectively (consistent and reversed, respectively). **(c)** Fractions of neurons with consistent and reversed regression coefficients for value. **p*=0.011, χ^2=6.3 (χ^2-test). (**d**) To determine whether neural responses to negative outcomes are related to visual responses to light, we recorded in three sessions dorsomedial striatal neuronal activity (n=28) while repeatedly delivering a light stimulus (with random 1–2 s durations) for 90 min without any reward delivery. Light-induced neural responses were greatly reduced within 30 min (spike density functions, σ=100 ms; shading, SEM), indicating that striatal neuronal responses during the outcome period in unrewarded trials are largely related to negative outcome rather than the light stimulus per se. (**e-h**) We also examined dorsomedial striatal neuronal activity (n=89, including 18 dSPNs and 14 iSPNs, each optogenetically identified) while delivering a light stimulus with and without respect to trial outcome. (**e**) Experimental scheme. The light stimulus was delivered not only at the onset of trial outcome in reward omission trials (early stimulus), but also 2.5 s after trial outcome in all trials (late stimulus). In this experiment, only one odor cue with a 50% reward probability was presented and the inter-trial interval was increased to 6–7 s. (**f**) A representative SPN showing elevated activity in response to the early light stimulus (negative trial outcome), but not to the late (control, arrows) light stimulus. (**g-h**) Group data. (**g**) Population responses (spike density functions, σ=100 ms) of 89 SPNs to the early and late light stimuli. (**h**) Population responses of 18 dSPNs and 14 iSPNs to the late light stimulus. As shown, the late (control) light stimulus induced no detectable changes in SPN activity.

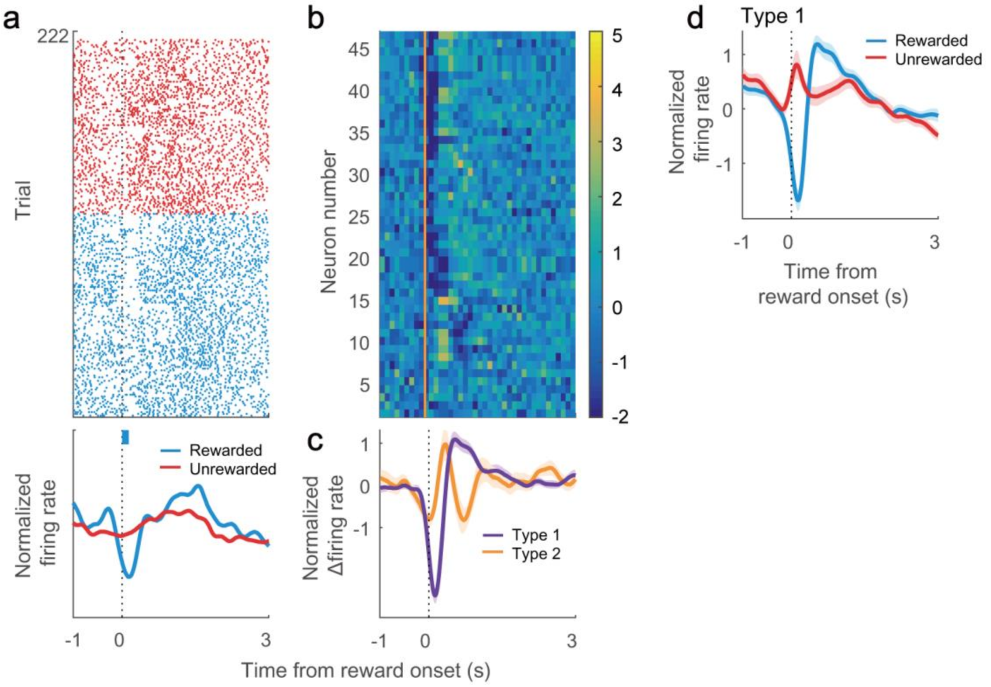

Supplementary Figure 6. Reponses of TANs to reward. (a) A representative example. A spike raster plot and a spike density function (σ=100 ms) for a TAN are shown. Blue, rewarded trials. Red, unrewarded trials. **(b)** Responses of all putative TANs (n=48) to reward are shown as z-normalized Δfiring rate. This follows the same format as Fig. 5a. These response patterns were classified into type 1 (inactivated by reward) and type 2 (activated by reward). (**c**) Mean responses (z-normalized Δfiring rate) of type 1 and type 2 TANs. (**d**) Mean z-normalized firing rates of type 1 TANs are shown separately for rewarded and unrewarded trials. Shading, SEM.

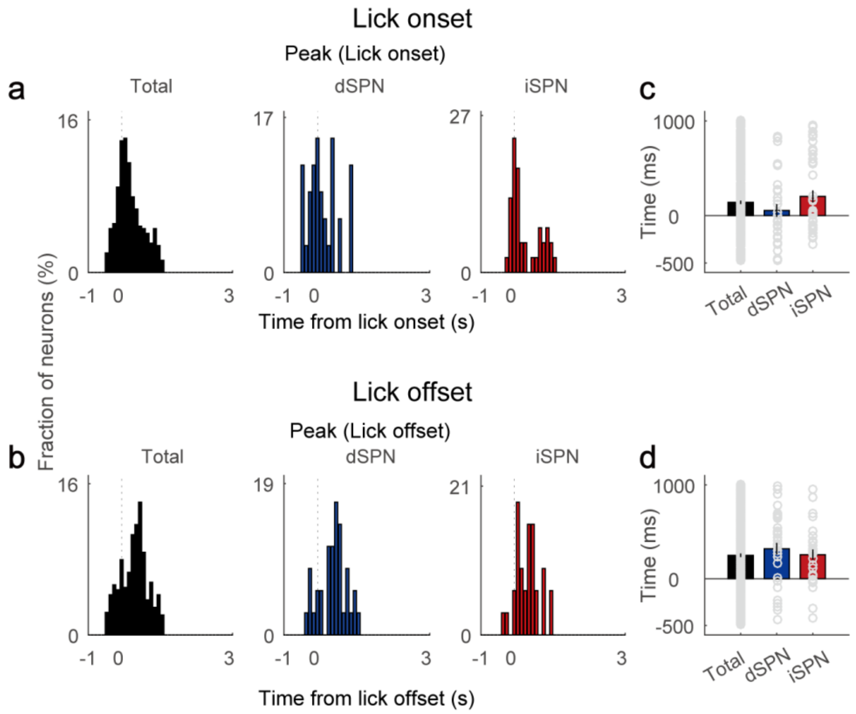

Supplementary Figure 7. Times of SPN responses to lick onset and offset. (**a, b**) Frequency histograms showing distributions of the timing of the peak neural response to lick onset (**a**) and offset (**b**). Shown are those SPNs with peak responses within 0.5 before and 1 s after lick onset or offset. (**c, d**) Mean peak times of the neural responses to lick onset (**c**) and offset (**d**). Total, all SPNs including untagged ones. Circles indicate individual neurons (Total, all SPNs including untagged ones, n=730, dSPN n=77, iSPN n=75).

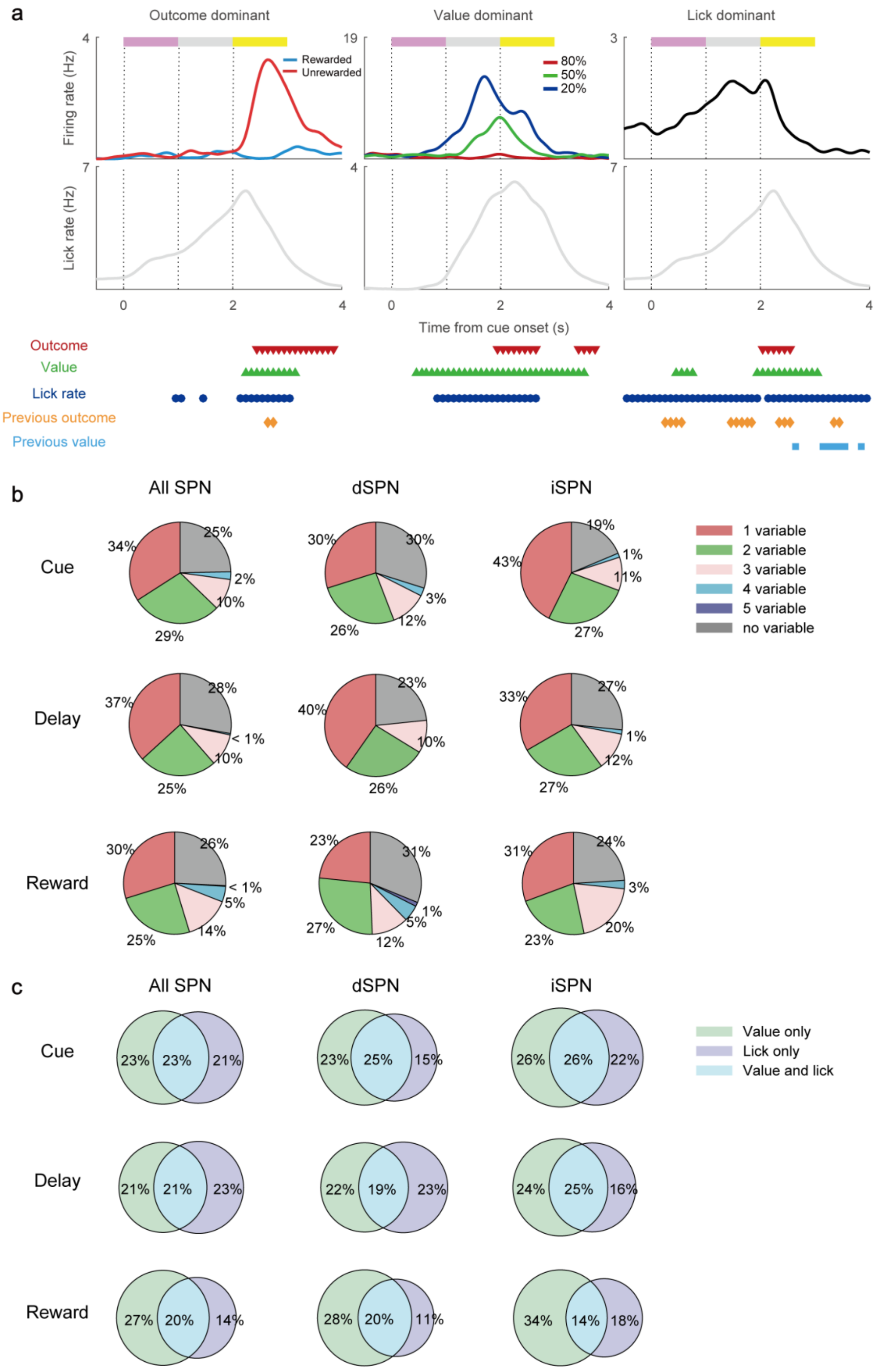
a
Outcome dominant
Value dominant
Lick dominant
Firing rate (Hz)
Lick rate (Hz)
Rewarded
Unrewarded
80%
50%
20%
Time from cue onset (s)
Outcome
Value
Lick rate
Previous outcome
Previous value
b
All SPN
dSPN
iSPN
Cue
Delay
Reward
1 variable
2 variable
3 variable
4 variable
5 variable
no variable
c
Value only
Lick only
Value and lick

Supplementary Figure 8. Overlapping representation of multiple task variables in the dorsomedial striatum. (a) Three representative SPNs responding to multiple task variables. The top panel presents spike density functions (σ=100 ms) grouped according to outcome (outcome dominant), value (value dominant), or lick rate (lick dominant). The middle panel shows mean lick rates during the task. In the bottom panel, each triangle indicates the time window during which neural activity was significantly modulated by outcome (red), value (green), lick rate (blue), previous outcome (orange), or previous value (cyan). **(b)** Proportions of all SPNs, dSPNs, and iSPNs coding different numbers of the five task variables (outcome, value, lick rate, previous outcome, and previous value) during the cue, delay, and reward periods (1 s each). **(c)** Proportions of SPNs responding to cue and/or lick rate during the cue, delay, and reward periods (1 s each).

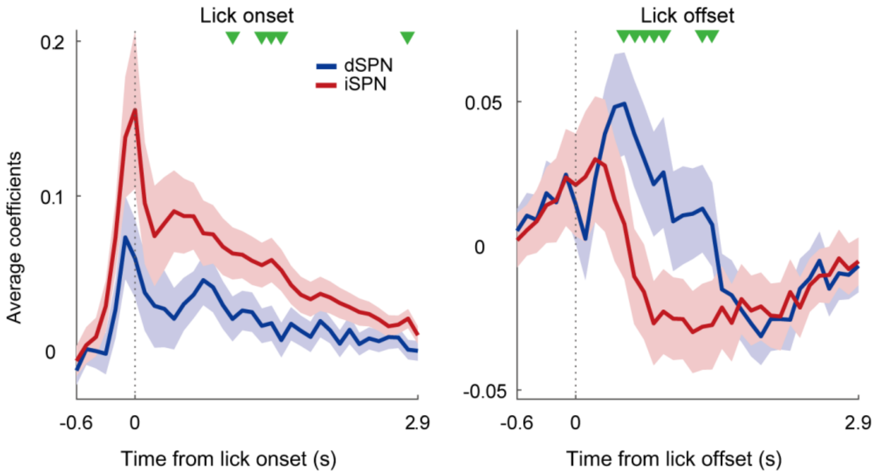

Supplementary Figure 9. Regression coefficients for lick onset and offset. Mean regression coefficients for lick onset (**a**) and offset (**b**) for dSPNs (n=77) and iSPNs (n=75) (*eq*. 3). Triangles denote significant differences between dSPNs and iSPNs (Wilcoxon's rank-sum test, $p < 0.05$). Shading, SEM.
